# Genomic investigation of a suspected *Klebsiella pneumoniae* outbreak in a neonatal care unit in sub-Saharan Africa

**DOI:** 10.1099/mgen.0.000703

**Published:** 2021-11-18

**Authors:** Jennifer Cornick, Patrick Musicha, Chikondi Peno, Ezgi Seager, Pui-Ying Iroh Tam, Sithembile Bilima, Aisleen Bennett, Neil Kennedy, Nicholas Feasey, Eva Heinz, Amy K. Cain

**Affiliations:** ^1^​ Malawi-Liverpool-Wellcome Trust Clinical Research Programme, Blantyre, Malawi; ^2^​ Institute of Infection, Veterinary and Ecological Sciences, University of Liverpool, Liverpool, UK; ^3^​ Mahidol-Oxford Tropical Medicine Research Unit, Mahidol University, Bangkok, Thailand; ^4^​ Centre for Tropical Medicine and Global Health Nuffield Department of Medicine, University of Oxford, Oxford, UK; ^5^​ Centre for Inflammation Research, Queen’s Medical Research Institute, University of Edinburgh, Edinburgh, UK; ^6^​ Birmingham Heartlands Hospital, University Hospitals Birmingham NHS Foundation Trust, Bordesley Green East, Birmingham, B9 5SS, UK; ^7^​ Liverpool School of Tropical Medicine, Liverpool, UK; ^8^​ Institute for Infection and Immunity, St George’s, University of London, London, UK; ^9^​ Department of Paediatrics, College of Medicine, University of Malawi, Zomba, Malawi; ^10^​ Centre for Medical Education, Queen’s University Belfast, Belfast, Ireland; ^11^​ Pathogen Genomics, Wellcome Sanger Institute, Cambridge, UK; ^12^​ Department of Molecular Sciences, Macquarie University, Sydney, Australia

**Keywords:** *Klebsiella pneumoniae*, hospital outbreak, antimicrobial resistance, neonatal infection, sub-Saharan Africa, genome sequencing

## Abstract

A special-care neonatal unit from a large public hospital in Malawi was noted as having more frequent, difficult-to-treat infections, and a suspected outbreak of multi-drug-resistant *Klebsiella pneumoniae* was investigated using genomic characterisation. All *K. pneumoniae* bloodstream infections (BSIs) from patients in the neonatal ward (*n*=62), and a subset of *K. pneumoniae* BSI isolates (*n*=38) from other paediatric wards in the hospital, collected over a 4 year period were studied. After whole genome sequencing, the strain sequence types (STs), plasmid types, virulence and resistance genes were identified. One ST340 clone, part of clonal complex 258 (CC258) and an ST that drives hospital outbreaks worldwide, harbouring numerous resistance genes and plasmids, was implicated as the likely cause of the outbreak. This study contributes molecular information necessary for tracking and characterizing this important hospital pathogen in sub-Saharan Africa.

## Data Summary

The *Klebsiella pneumoniae* genome sequences completed using an Illumina platform from this study are deposited in GenBank under project no. PRJNA641987 (https://www.ncbi.nlm.nih.gov/bioproject/?term=PRJNA641987) and the European Nucleotide Archive under project no. PRJEB19322 (https://www.ebi.ac.uk/ena/browser/view/PRJEB19322). The previously published *K. pneumoniae* sequences used as benchmarks of global context can be found in the European Nucleotide Archive under accession no. ERP000165.

*Klebsiella pneumoniae* is an opportunistic pathogen responsible for an increasing burden of hospital-acquired infections globally. It readily acquires antimicrobial resistance determinants via mobile genetic elements and plasmids [1], with particular sequence types (STs), such as those from clonal complex (CC) 258 (ST258, ST11), ST14/15 and ST405 reported to cause a large proportion of cephalosporin- and carbapenem-resistant infections with high mortality [2]. Critically, sepsis caused by multi-drug-resistant (MDR) *K. pneumoniae* is a growing challenge in neonatal care units; one systematic review of extended spectrum beta-lactamase (ESBL)-producing *Enterobacteriaceae* in neonatal care units reported that ESBL *K. pneumoniae* is the pathogen most frequently responsible for outbreaks in these settings, and is associated with a mortality rate of 31 % [3]. There are multiple reports detailing the prevalence and genetic epidemiology of MDR *K. pneumoniae* causing hospital outbreaks in high-income settings [2, 4, 5]. However, reports from low- and middle-income settings, where such data are of immense value in implementing appropriate infection prevention control and treatment regimes, are scarce.

**Fig. 1.**
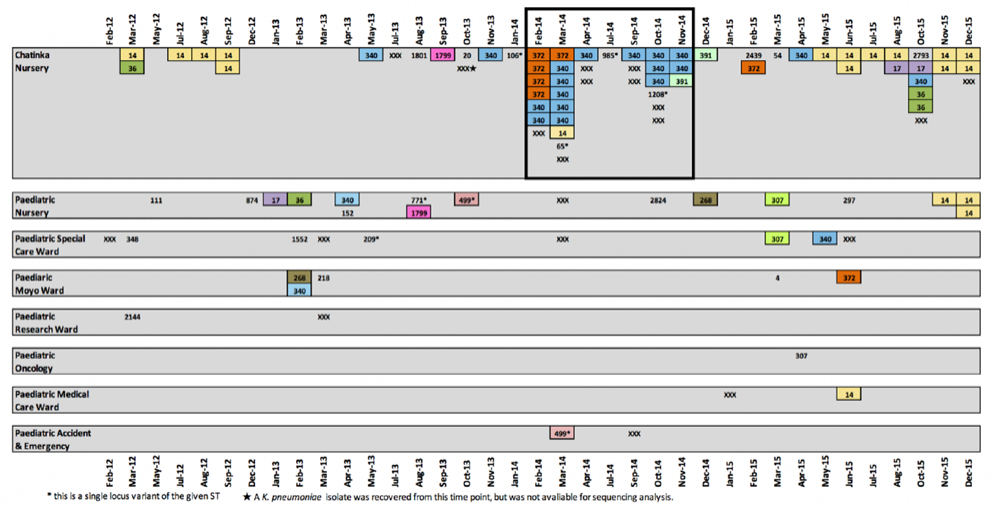
Schematic showing the location, sequence type (ST) and date of isolation of the *K. pneumoniae* isolates that were subjected to whole genome sequencing (*n*=86). STs that were identified more than once in the dataset are highlighted with a coloured box reflecting the STs. Singleton STs are not highlighted. Non-viable isolates (*n*=4) and those that failed sequencing (*n*=14) but were earlier confirmed as *K. pneumoniae* by the diagnostic laboratory are marked on the schematic as ‘XXX’ in order to give a complete picture of the number of BSI *K. pneumoniae* cases reported from Chatinkha each month during the study period. Months where no cases were reported are omitted from the schematic. The suspected outbreak period is highlighted with a black outline.

**Fig. 2.**
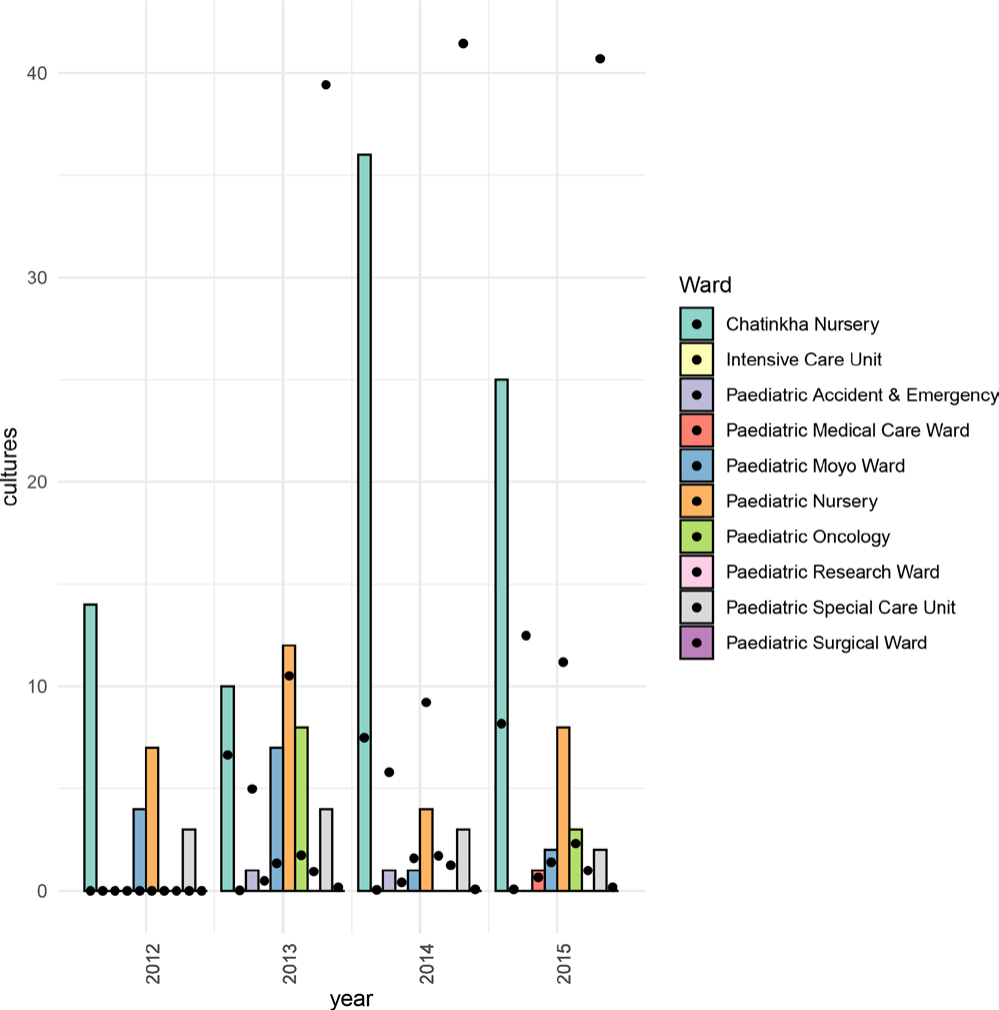
Bar chart showing the number of *K. pneumoniae *-positive blood cultures for Chatinkha ward and other paediatric wards at QECH. The chart is overlaid with a scatter plot showing the total number of all blood cultures (including those positive for any bacteria and those that were culture-negative) taken from the paediatric wards at QECH, Malawi, from 2012 to 2015 divided by 100. Data on the total number of blood cultures taken were not available for 2012 and are not shown.

Queen Elizabeth Central Hospital (QECH) is the referral hospital in southern Malawi and care is free at the point of delivery. QECH serves both as district hospital for Blantyre (population 900 000) and is the tertiary referral hospital for the surrounding districts. QECH admits approximately 10 000 adult (aged ≥16 years) and 30 000 paediatric (aged <16 years) medical patients annually. Chatinkha is a 70-bed neonatal special care unit in QECH. Given that Chatinkha predominantly admits neonates born at QECH with complications, the majority of blood stream infections (BSIs) diagnosed on this unit are considered to be hospital-acquired. At QECH, all children with clinically suspected sepsis undergo a blood culture (BC) test as previously described [6]. The BC service typically identifies fewer than two *K *. *pneumoniae* BSI on Chatinkha each month, but a spike in *K. pneumoniae* isolates was observed in February (*n*=7) and March (*n*=9) 2014. The number of cases temporarily decreased, yet September to November 2014 showed a second peak in cases (Fig. 1). Despite Chatinkha having the smallest capacity of all of the paediatric wards at QECH, from February to November 2014 over 75 % of all paediatric *K. pneumoniae* BSI (*n*=33/43) were from neonates admitted to Chatinkha. This rise was not due to an increase in the number of blood cultures taken, which remained stable (Fig. 2). Furthermore, *K. pneumoniae* BSIs in Chatinkha during this period were untreatable with locally available antibiotics (most commonly penicillin, gentamicin, cotrimoxazole; less commonly ciprofloxacin, ceftriaxone; carbapenems unavailable); most displayed identical antimicrobial resistance patterns, suggesting the possibility of a clonal outbreak.

Here, we performed a genomic investigation into the suspected *K. pneumoniae* outbreak, sequencing genomes from February to November 2014 (boxed in Fig. 1) and all viable, archived *K. pneumoniae* BSI isolates from Chatinkha (*n*=62) and a subset of *K. pneumoniae* BSI isolates (*n*=38) from other paediatric wards, isolated from January 2012 to December 2015. Patient records were retrospectively analysed to establish mortality outcomes. Blood culture collection, processing and antimicrobial susceptibility testing methods, based on the disc diffusion method and BSAC guidelines, for isolates at QECH have been previously described [6].

All DNA samples (*n*=100) were extacted with a Wizard Genomic DNA Purification Kit (Promega) and subjected to whole genome sequencing using the Illumina HiSeq X-Ten platform at the Wellcome Sanger Centre, UK, and the Oxford Genomics Centre. Fourteen sequenced genomes failed initial quality control using Kraken (more than 5 % of a species not part of the *K. pneumoniae* species complex assigned) [7]; the remaining 86 samples (Chatinkha *n*=56, other wards *n*=30) yielded, on average, 2.3 million reads per sample and an average of 77 contigs (range 22–259). The sequences were deposited in the European Nucleotide Archive (ENA) under project number PRJNA641987 and NCBI under project number PRJEB19322.

Multilocus sequence (MLST) typing was performed *in silico* as described elsewhere [8]. Resistance genes were identified using SRST2 v0.2.0[9] (https://github.com/katholt/srst2), plasmids using *in silico* PCR-based replicon typing [10] and PlasmidFinder version 2.0.1[11], capsular and O-antigen types using Kaptive (version 0.5.1; https://github.com/katholt/Kaptive) [12] and virulence genes using Kleborate v0.3.0 (https://github.com/katholt/Kleborate) [13, 14]. In order to place the study dataset within a global context, we compared our genomes to a previously published dataset from an international *K. pneumoniae* study [2]. A core gene alignment of the combined genomic dataset was generated using roary [15] and, from this, single nucleotide variants were used to generate a phylogeny with RaxML v.7.8.6 [16].

Whole genome sequencing analysis identified two lineages, ST340 and ST14, as the dominant *K. pneumoniae* STs recovered from neonates admitted to Chatinkha (Fig. 1).

**Impact Statement**

Multi-drug-resistant *Klebsiella pneumoniae* is an important nosocomial pathogen that has been flagged by the World Health Organization and the Centre for Disease Control as an urgent threat to human health. Despite the importance of this pathogen and its worldwide distribution, the reporting and tracking of *K. pneumoniae* across sub-Saharan Africa is limited, and even less so from Malawi, especially using genomic data. In this study, we investigate an outbreak of extremely multiply resistant *K. pneumoniae* from a neonatal ward in a large public hospital in southern Malawi, using whole genome sequencing, across a 4 year period (2012–2015). We analysed the genomes of the isolates using various approaches to understand the phylogenetic typing features, antibiotic resistance and virulence properties and compared these with sequenced strains from other paedeatric wards in the hospital and put them in a global context. This study helps to understand the optimal local treatment options available and establish the virulent strain types of this important pathogen that are circulating in Malawi as well as adding information to the critical global network of *K. pneumoniae* genomic data.

## There was a discrete outbreak of MDR ST340

**Fig. 3.**
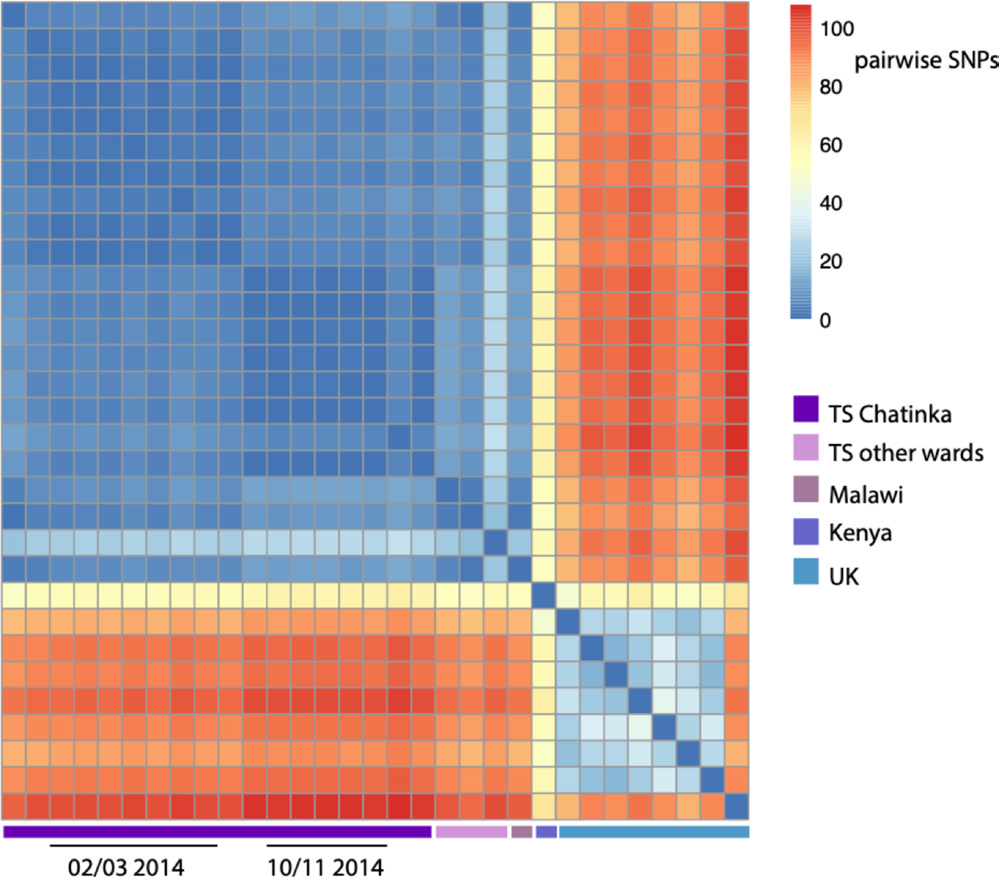
Heatmap showing the number of SNP differences between the *K. pneumoniae* ST340 isolates sequenced as part of the outbreak investigation. To bring the relatedness of our samples into context with other ST340 isolates, the analysis includes a community-acquired Malawian ST340 sequenced as part of a previous study at QECH [6], a single ST340 isolate from a study in Kenya [20] and eight ST340 genomes from the UK [21].

**Fig. 4.**
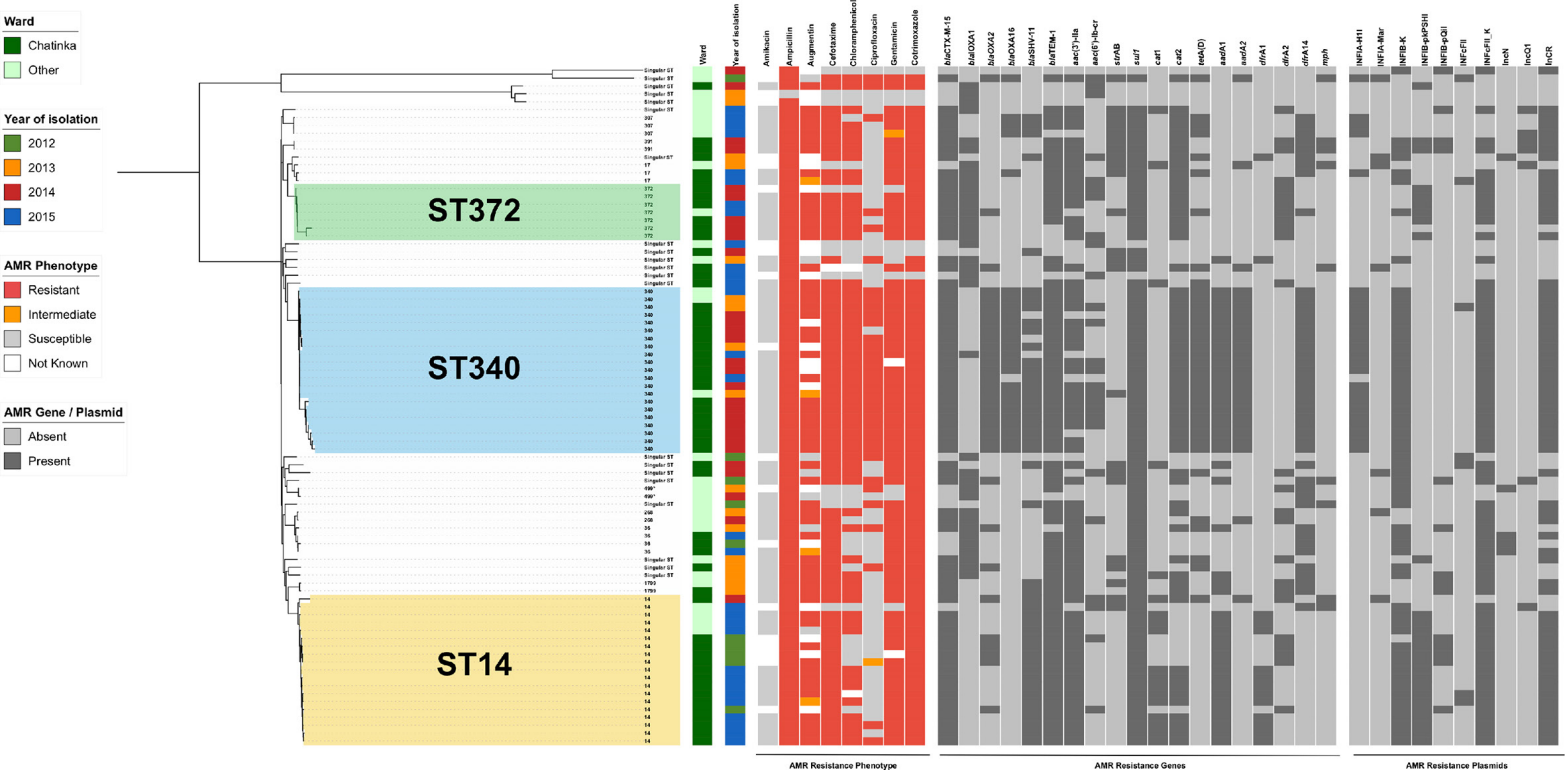
The distribution of antimicrobial resistance elements within the *K. pneumoniae* isolates from QECH. The phylogeny is based on a core genome SNP alignment of the Malawian isolates (*n*=86), and the branches are labelled with the ST. The panel adjoining the phylogeny shows the antimicrobial resistance phenotype and the absence/presence of key antimicrobial resistance genes and plasmids amongst the isolates. Beta-lactamase genes: *bla*_CTXM_, *bla*_TEM_, *bla*_OXA_, *bla*_SHV_. Quinolone resistance genes: *aac3, aac6*. Streptomycin resistance gene: *strAB*. Sulphonamide resistance gene: *sul1*. Chloramphenicol resistance genes: *cat1*, *cat2*. Tetracycline resistance gene: *tetA*. Aminoglycoside resistance genes: *aadA, aad1*. Trimethoprim resistance genes: *dfrA1, dfrA2, dfrA14*. Macrolide resistance gene: *mph*.

ST340, an ST within CC258 that has been associated with MDR hospital infections worldwide [17, 18], accounted for almost a third (30%, *n*=17/56) of all *K. pneumoniae* isolated from Chatinkha during the study period. All ST340 isolates were capsular type 15 and serotype O4 and differed from one another by ≤30 SNPs (Fig. 3), indicating that a single ST340 lineage was circulating in the ward over the entire study period. Over the suspected outbreak period, ST340 accounted for more than half of the *K. pneumoniae* BSIs from Chatinkha (58%, *n*=14/24), strongly suggesting that dissemination of this clonal strain within Chatinkha was responsible for the peak in BSIs observed from February to November 2014. The ST340 isolates were resistant to augmentin, ampicillin, ceftriaxone, chloramphenicol, ciprofloxacin, cotrimoxazole and gentamicin and susceptible only to amikacin, an antibiotic not locally available. Consistent with this, all ST340 isolates harboured multiple antibiotic resistance genes, conserved between all ST340 isolates, namely *bla*_CTX-M-15_, *bla*_OXA-1_, *bla*_OXA-2_, *bla*_OXA-16_, *bla*_SHV-11_, *bla*_TEM-1_ (beta-lactams), *aac*(6')-Ib-cr (quinolone), *str*AB (streptomycin), *sul1* (sulphonamides), *catA1, catA2* (phenicols), *tetA*(D) (tetracycline), *aadA1, aadA2*, *aac*(3’)-IIa (aminoglycosides), *dfrA1, dfrA2* (trimethoprim) and *mph* (macrolides), and a number of plasmids, including IncR and IncF types (see Fig. 4). Of the ST340 BSI cases reported in neonates on Chatinkha, outcome data were available for 13, four of whom died, bringing the case fatality rate to 31%, which is equivalent to other reports of MDR *K. pneumoniae* [2].

Prior to the outbreak, ST340 BSIs had only been isolated from Chatinkha on two previous occasions (May and November 2013) (Fig. 1). These isolates differed from the first two ST340 cases reported in February 2014 by fewer than five SNPs (Fig. 3), confirming that closely related strains were circulating in Chatinkha at least 6 months prior to the outbreak. Previous to this, ST340 was isolated on a single occasion from two other paedeatric wards, the earliest in February 2013. These two ST340s isolates differed from the presumed outbreak ST340 precursor isolate by fewer than seven SNPs. Whilst we cannot confirm the exact date at which ST340 was seeded into QECH, this indicates that ST340 was circulating in the hospital at least a year prior to the outbreak. Following the outbreak, ST340 was identified twice more on Chatinkha: a single case in April 2015 and again a single case in October 2015. October 2015 saw six cases of *K. pneumoniae* BSIs identified on Chatinkha, hinting at the start of another outbreak, but these six cases were caused by five different STs. The fact that ST340 continued to circulate in the ward after November 2014 but did not to contribute to a further peak in cases suggests that the success of this clone during the outbreak period was not driven by genomic factors alone.

Interestingly, a recent study characterizing MDR *Enterobacteriaceae *, using PFGE and PCR, across northern and central Malawi, identified seven isolates of ST340 *K. pneumoniae* in 2016–2017 [19]. This indicates that although MDR ST340 strains appeared to be circulating across the whole country during this time period, these strains were probably not identifical clones of the outbreak in QECH because PCR confirmed that the nothern isolates carried different resistance genes (eg *bla*_KPC-2_).

## ST14 did not contribute to the outbreak

Despite being the second most commonly isolated ST from Chatinkha (27%, *n*=15/56) during the study period, only a single ST14 case was reported during the outbreak period, in March 2014. ST14 showed a greater level of variation in their resistance profiles relative to ST340. These isolates were predominantly susceptible to locally available antibiotics (chloramphenicol and ciprofloxacin) in addition to amikacin and, thus, the ST14 infections would have been treatable (Fig. 4).

## ST372 related to a peak in cases

ST372 (capsular type: 43, serotype: O2V1) also appeared to contribute to the peak in *K. pneumoniae* BSIs over the outbreak period as ST372 was responsible for 20.8 % (*n*=5/24) of cases. ST372 was first observed in February 2014 in Chatinkha and caused four BSI in 1 month and the remaining five *K. pneumoniae* BSIs reported during the outbreak period belonged to five different STs. ST372 isolates displayed relatively disparate resistance and plasmid profiles and may not be clonal (Fig. 4).

## The outbreak strains in a global context

**Fig. 5.**
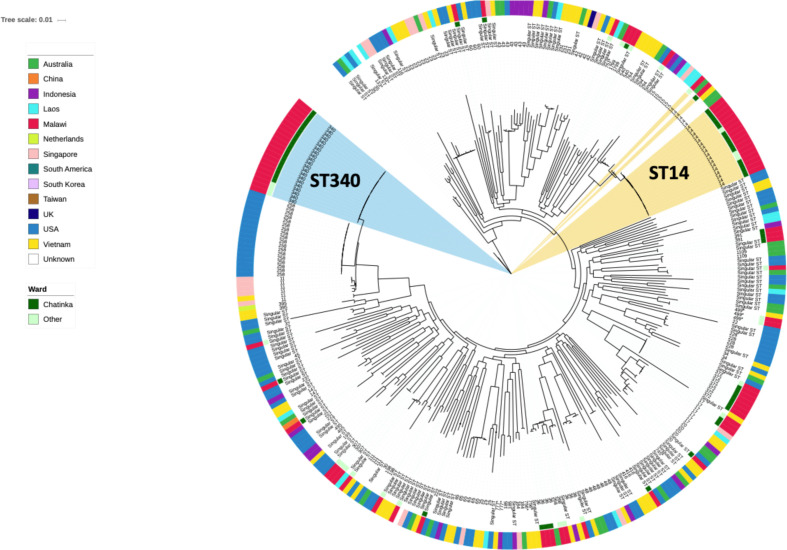
Population structure of *K. pneumoniae *. A core genome phylogeny of the Malawian KP-I isolates (*n*=81) in the context of a previously published global dataset. Branch labels are annotated with ST. The inner coloured circle indicates if the Malawian isolates were recovered from the Chatinkha neonatal care unit, while the outer ring indicates the country of isolation.

A core phylogeny was constructed to place isolates into a global context using a sequencing dataset of diverse *K. pneumoniae* isolates [2] (Fig. 5). Although no ST340 isolates were present in this dataset, the Malawian ST340 isolates were most closely related to ST258 isolates from the USA. Interestingly, ST14 strains from Malawi were clonally related (<100 SNPs) to international isolates with >99.9 % identity to isolates from Australia and the Netherlands, indicating a global spread. All ST372 isolates assayed in this study were from Malawi and we were unable to place them in a global context as no comparative international strains were available in this dataset.

## Conclusion

We studied *K. pneumoniae* BSIs isolated from neonates admitted to a neonatal unit over a 4 year period, encompassing a suspected outbreak in 2014, as well as a representative subset of *K. pneumoniae* BSI reported from other paediatric wards within the same hospital.

We show that ST340, and to a lesser extent ST372, caused an increase in BSIs reported on the neonatal ward in 2014. ST340 was observed in other wards prior to the outbreak, suggesting that it was circulating in the hospital prior to the outbreak. ST372 was not identified in other wards prior to the outbreak, but this may be due to the limitations of our sampling. In addition to the outbreak lineages, we observed a large cluster of ST14 isolates, which intermittently contributed to no more than two BSI cases per month on Chatinkha. All other STs identified in Chatinkha over the sampling period were only observed in sporadic single isolate clusters. From the sequencing analysis alone, it is unclear what exact factors allowed the specific lineages to persist or successfully transmit within Chatinkha beyond antibiotic selection and this is under further investigation by performing genomics of environmental samples and staff swabs. Furthermore, continual monitoring of the hospital environment, especially for the re-emergence of MDR ST340, is urgently needed in order to implement measures to prevent the persistence and/or spread of these locally untreatable lineages.
